# Ultrasound Plus Vacuum-System-Assisted Biocatalytic Synthesis of Octyl Cinnamate and Response Surface Methodology Optimization

**DOI:** 10.3390/molecules27217148

**Published:** 2022-10-22

**Authors:** Ming-Fang Tsai, Shang-Ming Huang, Hsin-Yi Huang, Shuo-Wen Tsai, Chia-Hung Kuo, Chwen-Jen Shieh

**Affiliations:** 1 Department of Food Science and Biotechnology, National Chung Hsing University, Taichung 402, Taiwan; 2 Biotechnology Center, National Chung Hsing University, Taichung 402, Taiwan; 3 Department of Nutrition, China Medical University, Taichung 406, Taiwan; 4 Department of Seafood Science, National Kaohsiung University of Science and Technology, Kaohsiung 811, Taiwan; 5 Center for Aquatic Products Inspection Service, National Kaohsiung University of Science and Technology, Kaohsiung 811, Taiwan

**Keywords:** lipase, cinnamate ester, response surface methodology, ultrasound, vacuum, transesterification

## Abstract

Cinnamic acid is one of the phenolic compounds that is isolated from cinnamon, or other natural plants, and has a wide range of physiological activities. However, the application of cinnamic acid is limited due to its poor solubility and low oral bioavailability. In this study, the feasibility of producing octyl cinnamate by ultrasonic assistance, combined with a rotary evaporation under vacuum, was studied using methyl cinnamate and octanol as the starting materials. A Box–Behnken design (BBD) was employed to evaluate the effects of the operation parameters, including reaction temperature (55–75 °C), reaction time (4–12 h), and ultrasonic power (90–150 W) on the production of octyl cinnamate. Meanwhile, the synthesis process was further optimized by the modeling response surface methodology (RSM). The data indicated that octyl cinnamate was efficiently synthesized from methyl cinnamate and octanol using the ultrasound plus vacuum system; further, this system was superior to the conventional method. According to the RSM model for the actual experiments, a reaction temperature of 74.6 °C, a reaction time of 11.1 h, and an ultrasound power of 150 W were determined to be the best conditions for the maximum molar conversion of octyl cinnamate (93.8%). In conclusion, the highly efficient synthesis of octyl cinnamate by a rotary evaporator with an ultrasound plus vacuum system was achieved via RSM optimization.

## 1. Introduction

Cinnamic acid, 3-phenyl-2-propenoic acid, is an organic compound with the structural formula C_6_H_5_CH=CHCOOH. Cinnamic acid occurs naturally in plants or materials and is one of the main phenolic compounds [1]. Cinnamic acid and its derivatives are phytochemical compounds with a wide range of biological effects. Several studies have reported that cinnamic acid displays antioxidant, antimicrobial, anticancer, neuroprotective, anti-inflammatory, and anti-diabetic properties [2,3]. However, due to the low bioavailability of cinnamic acid and its derivatives, which leads to limited intestinal absorption [1], improvements in the entrapment of phenolic acids are now emphasized. According to previous studies, the bioavailability of phenolic acids can be enhanced by esterification, which increases their lipophilic properties for use in drugs [4,5]. In addition, esters are insoluble in water, many organic sunscreens are also composed of esters, which can increase the water resistance of sunscreen products and enhance the sunscreen effect.

In general, the chemical synthesis of esters has several disadvantages, such as non-specific reactions, long reaction times, many byproducts, environmental pollution, etc. [6,7,8]. In addition, the temperature and pH can cause a tendency for phenolic esters to oxidize, which makes them difficult to synthesize by chemical methods [9]. In recent years, products synthesized via biocatalysis can be identical to natural products, which has made the application of biocatalysis in ester synthesis become more popular [10]. Enzymatic catalysis is carried out under moderated reaction conditions (pH, temperature, and atmospheric pressure), and the substrate is more regulated, allowing the production of high-quality natural products [11,12]. The advantages of biocatalysis are that they are suitable for industrial use, i.e., in their high specificity, selectivity, low energy consumption, and high yield [13,14].

As a biocatalyst, lipase can not only hydrolyze triglycerides [15,16], but also catalyze the reverse synthesis of esters under certain conditions [17,18]. A previous study used the immobilized lipase Novozym^®^ 435 to esterify cinnamic acid with an alcohol in organic solvent media. The results showed that more hydrophobic solvent mixtures and lower water activity improved enzyme activity and bioconversion [19]. In addition, compounds can be obtained via lipase-catalyzed esterification, deamination reaction, or in the isolation of optically active racemates under organically solvent conditions [20,21]. However, some biocatalytic reactions still require long reaction times in order to achieve the desired results. Several studies have shown that ultrasonic or vacuum operation contribute to the efficiency of esterification that is catalyzed by immobilized lipases, such as retinyl laurate [22], phenylethyl ester [23], 4′-acetoxyresveratrol [24], ethyl butyrate [25], and D-isoascorbyl palmitate [26]. Nowadays, ultrasound has been widely used for the extraction, emulsification, and chemical and/or enzymatic synthesis of compounds [27,28,29]. Although ultrasound-assisted biocatalysis is an effective method to shorten the reaction time, excessive substrate or formation of hydrophilic byproducts can cause enzyme inhibition or produce a hydrophilic hindrance layer in lipase-catalyzed reaction, thus reducing the lipase activity [30,31]. Further, technical difficulties arise in practice due to the steric hindrance of long-chain substrates, which increases the time to complete the synthetic reaction. To overcome these problems, an eco-friendly process for the synthesis of octyl cinnamate using ultrasonic irradiation, combined with a rotary evaporator system under vacuum condition was used in this paper.

In this study, the synthesis of octyl cinnamate by transesterification of methyl cinnamate and octanol was investigated using an immobilized lipase (Novozym^®^ 435). Ultrasonic irradiation combined with a rotary evaporator system was used to shorten the reaction time and remove byproducts in order to avoid reducing the enzyme activity. Additionally, reaction parameters affecting the synthesis of octyl cinnamate were evaluated. The optimal synthesis conditions of octyl cinnamate were modeled by a response surface methodology (RSM)—which used a three-level–three-factor Box–Behnken design—including the effects of reaction time, reaction temperature, and ultrasonic power in regard to the molar conversion of octyl cinnamate.

## 2. Results and Discussion

### 2.1. Effect of Ultrasound Plus Vacuum

Esterification of cinnamic acid can increase the stability, solubility/lipophilicity, and antioxidant effect of oil-based formulations, and it is also more suitable for human absorption in terms of physiological activity. Several studies have been attempted regarding the lipase-catalyzed modification of cinnamic acid derivatives with fatty acids or triacylglycerols, but it takes a long time to obtain synthetic products [32]. The hydrophilic character of phenolic acids has been reported to reduce their antioxidant effect in inhibiting the autoxidation of fats and oils. To modify their solubility in oil-based formulations and emulsions, esterification of phenolic acid is one of the solutions [33]. Recently, the synthesis of 3,4-dimethoxycinnamoylated phospholipids via lipase-catalyzed interesterification of egg-yolk phosphatidylcholine with the ethyl ester of 3,4-dimethoxycinnamic acid has been developed [34]. Lue et al. optimized the esterification of cinnamic acid and oleyl alcohol by the use of immobilized lipase Novozym^®^ 435 in organic solvent media [19]. However, the process takes 12 days to complete the reaction. 

**Scheme 1:**
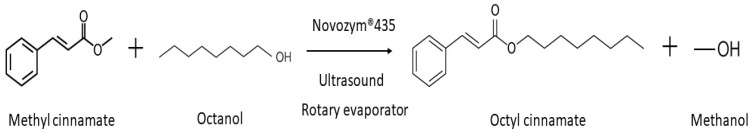
Ultrasonic irradiation plus vacuum system for production of octyl cinnamate.

Hence, a new synthetic route for the synthesis of octyl cinnamate in a solvent-free environment was attempted in this study. The transesterification reaction of methyl cinnamate with octanol catalyzed by lipase was used for the synthesis of octyl cinnamate, as shown in Scheme 1. 

**Figure 1:**
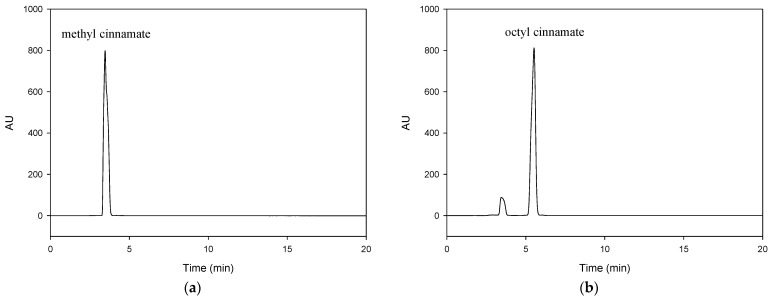
HPLC analysis of the reaction mixture (**a**) before and (**b**) after lipase-catalyzed transesterification reaction.

In order to perform lipase-catalyzed reactions without the use of organic solvents, only the reactants need to be mixed in non-aqueous, ultrasonic, and vacuum systems. Solvent-free systems are those in which the reactants are used as solvents in the reaction, which can greatly increase the reaction rate [35,36,37]. In this case, octanol was used as a reactant and solvent. The reaction was performed using a vacuum rotary evaporator and the flask was continuously rotated with a vacuum in a constant-temperature water bath. After the reaction, the reaction product was analyzed using an HPLC chromatogram with a UV detector at 307 nm. The reactant and reaction product are shown in Figure 1a,b, respectively. Methyl cinnamate had a shorter retention time (3.5 min) in the C18 column, while the reaction product, octyl cinnamate, had a longer retention time (5.5 min) due to octyl group increasing its hydrophobicity.

**Figure 2:**
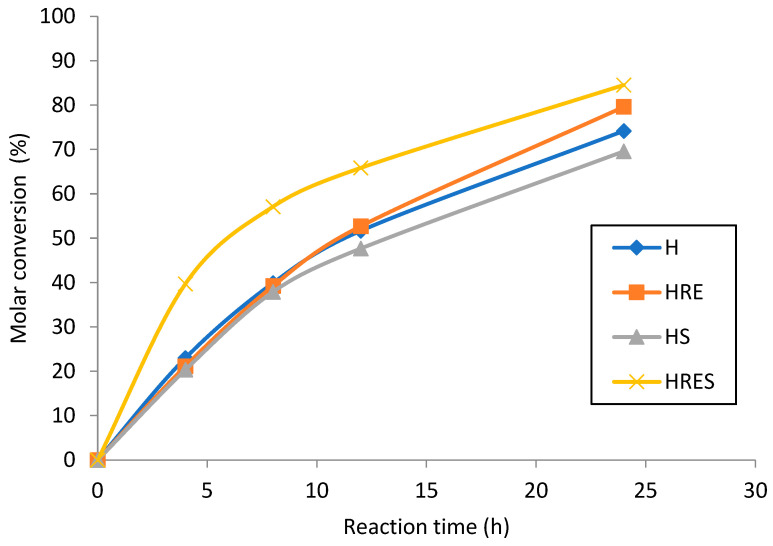
Effect of equipment on the molar conversion of octyl cinnamate. H: rotary evaporator without vacuum; HRE: rotary evaporator with vacuum; HS: ultrasonic bath; and HRES: rotary evaporator with vacuum and ultrasonic bath. The ultrasonic bath was operated at 150 W.

This study is the first reported ultrasound plus vacuum system for biocatalytic synthesis of octyl cinnamate. The effect of various synthetic equipment on the conversion of octyl cinnamate was compared. Considering the reaction volume, the enzyme amount of 5000 PLU was employed in order to avoid the influence of the enzyme amount. The effect of ultrasound plus vacuum on the lipase-catalyzed synthesis of octyl cinnamate at a reaction temperature of 55 °C, and a substrate concentration of 1 mM are shown in Figure 2. The molar conversions of H, HRE, and HS were only about 20% after 4 h of reaction, but the molar conversion of HRES was 40%. The result showed that under the condition of ultrasound plus vacuum-assisted biosynthesis, not only was the molar conversion of octyl cinnamate increased within 4 h, but the highest conversion was also reached after 24 h. This is due to the lipase-catalyzed synthesis of octyl cinnamate using a rotary evaporator with a vacuum, which creates a negative pressure environment that helps to remove the formed methanol and thus shifts the reaction equilibrium towards the synthetic path in Scheme 1. It has been reported that the osmotic dehydration at vacuum had higher mass transfer kinetic constants and dehydration efficiency indices than it would at atmospheric pressure [38]. Hong et al. found the esterification conversion of triacylglycerol increased with an increasing vacuum [39]. Lee et al. also demonstrated that a vacuum can be used to control the content of water—a byproduct of the esterification reaction—in order to improve the conversion of disononyl adipate [40]. In addition, many studies have also shown that ultrasound can be effective in enhancing lipase-catalyzed reactions [41,42]. Ultrasound is one of the green chemical synthesis techniques that has been successfully applied in organic chemistry [43,44]. Ultrasound contributes to high-frequency vibrations that cause cavitation in the liquids. Due to the high penetration effect, it can transmit powerful energy [45]. The energy increases the bonding probability when the substrate and enzymes interact. It has been reported that lipase-catalyzed transesterification with ultrasound had a higher external mass transfer coefficient and a lower substrate concentration at the external surface of the immobilized enzyme [46]. After comparing the results of various equipment, HRES showed the most efficient and highest conversion rate; as such, this system was selected to study the optimization of reaction conditions.

### 2.2. Prime Experiment

**Figure 3:**
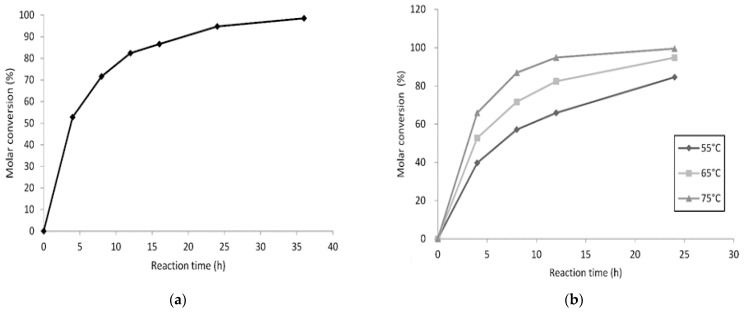
Effects of (**a**) reaction time, and (**b**) temperature, on the molar conversion of octyl cinnamate.

For the experimental design of the response surface methodology, preliminary tests were carried out using the rotary evaporator with vacuum and ultrasonic bath at a reaction temperature of 65 °C, a substrate concentration of 1 mM, and an enzyme amount of 5000 PLU (0.5 g). The results showed that the molar conversion of octyl cinnamate increased with increasing the reaction time. A value of 82.4% molar conversion was reached after 12 h; further, equilibrium was reached after 24 h (Figure 3a). Figure 3b shows the effect of reaction temperature on the molar conversion of octyl cinnamate, indicating that the molecular conversion of octyl cinnamate increased with an increase in reaction temperature. The lipase-catalyzed reaction at different reaction temperatures produced a significant effect on the synthesis of octyl cinnamate within 4 h. The highest molar conversion of octyl cinnamate was 93.9% when the reaction temperature was 75 °C and the reaction time was 12 h.

### 2.3. Experimental Design and Model Fitting

In the present study, a three-level–three-factor Box–Behnken design was employed to optimize the enzymatic synthesis of octyl cinnamate. Based on the preliminary experimental results (Table 1), the variables chosen for this study were reaction time (4–12 h), reaction temperature (55–75 °C), and ultrasonic power (90–150 W). Table 1 also shows the actual yields obtained in the experiments. Among the different treatments, the highest molar conversion (93.5 ± 2.1%) was obtained in treatment 4 (time 12 h, temperature 75 °C, and ultrasonic power 120 W), and the lowest molar conversion (35.5 ± 0.1%) was obtained in treatment 1 (time 4 h, temperature 55 °C, and ultrasonic power 120 W). The manipulated and response variables were analyzed to fit a regression model. The second-order polynomial equation obtained was as follows:

*Y* = −45.3866 + 1.311*X*_1_ + 11.22625*X*_2_ − 0.46767*X*_3_ + 0.0045625*X*_1_^2^ − 0.3782*X*_2_^2^ + 0.00116806*X*_3_^2^ − 0.061625*X*_1_*X*_2_ − 0.000391667*X*_1_*X*_3_ + 0.023167*X*_2_*X*_3_ (1)

**Figure 4:**
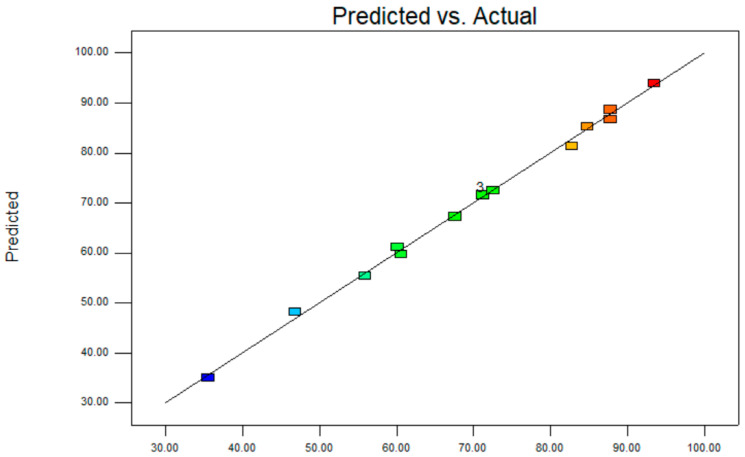
The relationship between the predicted and actual conversion of octyl cinnamate.

The analysis of variance is shown in Table 2. The results showed that the regression model fully reflected the actual relationship between the responses and the significant variables (*R*^2^ = 0.997). The coefficient of determination (*R*^2^) is the percentage of the variation that can be explained by the regression model in regard to the total variation. In this case, the *R*^2^ is close to 1, indicating that the developed model is well adapted. Figure 4 shows the predicted and actual conversion of octyl cinnamate. The adjusted coefficient of determination (Adj. *R*^2^ = 0.992) is also very high, indicating the high significance of the model. A “*p* > *F*” value of less than 0.05 indicates that the model (*p* < 0.0001) is significant. In this case, the linear terms of temperature (*X*_1_, *p* < 0.0001) and time (*X*_2_, *p* < 0.0001) provided a significant positive effect on the production of octyl cinnamate. The quadratic term of time (*X*_2_^2^, *p* = 0.0003) was also statistically significant. In addition, interaction terms of temperature × time (*X*_1_ × *X*_2_; *p* = 0.0129) and time × power (*X*_2_ × *X*_3_; *p* = 0.0080) were also observed.

### 2.4. Mutual Effect of Parameters

**Figure 5:**
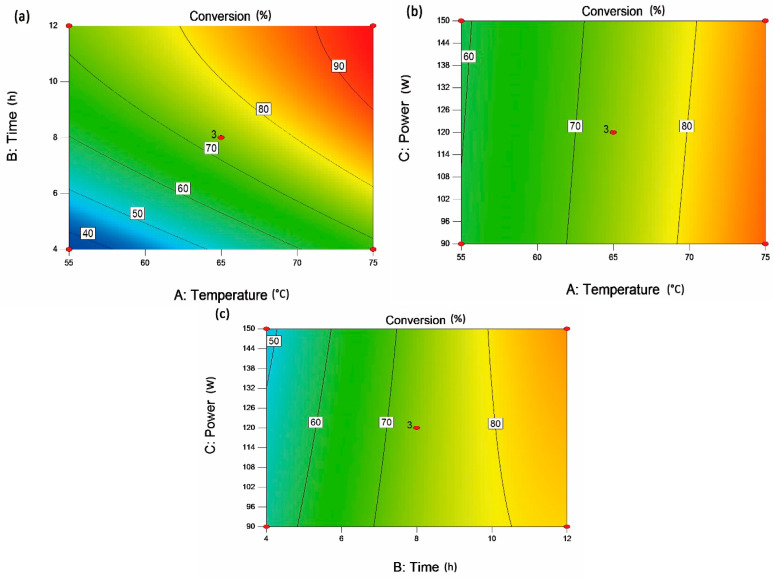
Contour plots show the different reaction behaviors (**a**) reaction temperature and reaction time with a fixed ultrasonic power of 120 W; (**b**) reaction temperature and ultrasonic power with a fixed reaction time of 8 h; and (**c**) reaction time and ultrasonic power with a fixed reaction temperature of 65 °C. The numbers on the contours indicate the molar conversion of octyl cinnamate under specific reaction conditions.

Figure 5 shows the effect of reaction temperature, time, and ultrasonic power on the molar conversion of octyl cinnamate. Figure 5a represents the effect of different reaction times and reaction temperatures on conversion at a fixed ultrasonic power of 120 W. At a low reaction temperature of 55 °C and a short reaction time of 4 h, the molar conversion was below 40%. The conversion increased with increasing reaction temperature and reaction time. At 75 °C and 12 h, the molar conversion reached very high levels (>90%). In contrast, the ultrasonic power has little effect on the molar conversion of octyl cinnamate. As shown in Figure 5b,c, the contour lines are almost parallel, indicating that the ultrasonic power has little effect on the molar conversion of octyl cinnamate. In the present study, both reaction time and reaction temperature have a significant effect on molar conversion. A similar compound commonly used as a UVB absorber, octyl methoxycinnamate, was synthesized by the esterification of *p*-methoxycinnamic acid with 2-ethyl hexanol using *Rhizopus oryzae* lipase, but it required 4 days to reach 91.3% conversion [47]. Vosmann et al. used lipase-catalyzed esterification in the solvent-free system to synthesize long-chain alkyl esters of *p*-methoxycinnamic acid, which in most cases required more than 72 h to achieve high conversion [48]. The longer the length of the alcohol the more it hinders substrate access to the active site of the enzyme, reducing the conversion rate of synthesis., e.g., the esterification of cinnamic acid with butanol and octanol obtained 73% and 55% yield after 7 days of reaction, respectively [49]. Hence, the use of ultrasound plus vacuum systems in lipase-catalyzed reactions can greatly reduce the reaction time and improve the synthetic conversion.

### 2.5. Attaining Optimum Conditions

The optimal conditions for the lipase-catalyzed synthesis of octyl cinnamate were predicted using the optimization function of the Design Expert Software. Table 3 shows the optimum condition for their experimental and predicted values. The maximum conversion of octyl cinnamate was obtained at 74.6 °C, 11.1 h, and 150 W, yielding 93.8% conversion. This experimental value is very close to the predicted value of the RSM model, which is 94.6%. The results indicate that RSM is an effective tool for modeling and optimizing the enzymatic synthesis process and that the rotary evaporator with a vacuum and ultrasonic bath can significantly reduce the reaction time of lipase-catalyzed synthesis of octyl cinnamate. The rotary evaporator with vacuum can evaporate the solvent to gas at temperatures below the boiling point of the solvent or even at room temperature. In this case, methanol, a byproduct of the reaction, can be evaporated during the reaction, thus allowing the reaction to proceed toward the synthetic pathway. Therefore, an ultrasound plus vacuum system can increase the conversion and shorten the reaction time. In addition, the Novozym^®^ 435 was used in an ultrasound-assisted packed-bed bioreactor operated continuously at a temperature of 73 °C for 7 days. The conversion was maintained between 93 and 97%, indicating that Novozym^®^ 435 showed stable activity for long-term operation [23]. In this study, Novozym^®^ 435 was repeatedly used at optimum condition and the conversion of octyl cinnamate was found to be more than 90%.

## 3. Materials and Methods

### 3.1. Materials

Immobilized lipase Novozym^®^ 435 (10000 PLU g^−1^; propyl laurate units)—from *Candida antarctica* B (EC 3.1.1.3), supported on a macroporous acrylic resin—was purchased from NovoNor disk Bioindustrials Inc. (Copenhagen, Denmark). Methyl cinnamate was purchased from Sigma Chemical Co. (St. Louis, MO, USA). Further, 1-octanol was purchased from Showa Chemical Industry Co., Ltd. (Tokyo, Japan). Molecular sieve 4 Å was obtained from Davison Chemical (Baltimore, MD, USA). All chemicals used were of analytical reagent grade.

### 3.2. Lipase-Catalyzed Synthesis of Octyl Cinnamate

All materials were dehydrated overnight through a 4 Å molecular sieve. Novozym^®^ 435 was used as a biocatalyst for the transesterification of methyl cinnamate with octanol (Scheme 1). Before the reaction, 1 mM (1.62 mg) of methyl cinnamate and 5000 PLU (0.5 g) of Novozym^®^ 435 were mixed thoroughly with octanol (10 mL) in a glass flask. Under different experimental conditions, a rotary evaporator (EYELA N-1100, Tokyo Rikakikai Co., Ltd., Tokyo, Japan) and a temperature-controlled ultrasonication water bath (Delta DC150H, New Taipei, Taiwan) were run at 80 rpm and 110 Torr for the emission-type glass flask.

### 3.3. Quantitation of Octyl Cinnamate

The reaction mixture of octyl cinnamate was analyzed by a high-performance liquid chromatography (HPLC). The 10-fold diluted sample was injected (20 µL) into an HPLC (Hitachi L-7400; Tokyo, Japan) equipped with a UV detector and an Inertsil ODS-3 column (5 µM, 250 mm × 4.6 mm). The isocratic elution was performed with 0.1% acetic acid and methanol at a flow rate set to 1 mL/min, and octyl cinnamate was detected under UV light at 307 nm. The integrated area of octyl cinnamate and methyl cinnamate in the HPLC chromatogram was used in order to calculate the molar conversion. The molar conversion was defined as: Peak area of octyl cinnamate per peak area of methyl cinnamate and octyl cinnamate × 100%.

### 3.4. Response Surface Methodology

A Box–Behnken design for 15 experimental runs was employed in this study. To avoid bias, the 15 experiments were performed in random order. The variables and levels of octyl cinnamate biosynthesis selected for this study were: reaction time (4–12 h), reaction temperature (55–75 °C), and ultrasonic power (90–150 W), which are coded as shown in Table 1. Each experimental point was carried out in duplicate. The experimental data (Table 1) were analyzed by the response surface regression procedure of Design-Expert (Version 8.0.6.1, Stat-Ease Inc., Minneapolis, MN, USA), resulting in a second-order polynomial equation (Equation (1)), as shown below:

*Y* = *β*_0_ + *β*_1_*X*_1_ + *β*_2_
*X*_2_ + *β*_3_*X*_3_ + *β*_12_*X*_1_*X*_2_ + *β*_13_*X*_1_*X*_3_ + *β*_23_*X*_2_*X*_3_ + *β*_11_*X*_1_^2^ + *β*_22_*X*_2_^2^ + *β*_33_*X*_3_^2^ (2)

where *Y* is an experimental response, *β*_0_ is a constant; *β*_1_, *β*_2_, and *β*_3_ are linear coefficients; *β*_11_, *β*_22_, and *β*_33_ are secondary effect coefficients; and *β*_12_, *β*_13_, and *β*_23_ are variable interaction coefficients. Further, *X*_1_ = temperature, *X*_2_ = time, and *X*_3_ = ultrasonic power.

## 4. Conclusions

In this study, an ultrasonic plus vacuum system was constructed to efficiently assist the immobilized lipase (Novozym^®^ 435) in the catalytic synthesis of octyl cinnamate. In this study, in order to model and optimize the biocatalysis, an RSM model based on the Box–Behnken design for the synthesis of octyl cinnamate was chosen, and the data were fitted and predicted. The high *R*^2^ value indicates that a lipase-catalyzed synthesis of octyl cinnamate via an ultrasound plus vacuum system was successfully established by the use of a Box–Behnken design and RSM. The optimal conditions were: reaction temperature 74.6 °C, reaction time 11.1 h, and ultrasonic power 150 W. The conversion of octyl cinnamate under these conditions was 93.8%, while the predicted data of the RSM model were 94.6%. In conclusion, the combination of a vacuum evaporation system and solvent-free reaction conditions can shorten the reaction time and reaction temperature for the synthesis of octyl cinnamate.

## Figures and Tables

**Table 1:** Box–Behnken design matrix and responses of dependent variables for the synthesis of octyl cinnamate.

Treatments ^a^	Independent Variable			Molar Conversion (%)		
	Temperature (°C)	Time (h)	Power (W)			
	*X*_1_	*X*_2_	*X*_3_	Actual Values ^b^	RSM Predicted Value	RSM Deviation
1	55 (−1) ^c^	4 (−1)	120 (0)	35.5 ± 0.1	35.1	0.5
2	75 (1)	4 (−1)	120 (0)	67.6 ± 0.3	67.3	0.4
3	55 (−1)	12 (1)	120 (0)	71.2 ± 0.3	71.6	0.4
4	75 (1)	12 (1)	120 (0)	93.5 ± 2.1	93.9	0.5
5	55 (−1)	8 (0)	90 (−1)	60.1 ± 0.2	61.1	1.0
6	75 (1)	8 (0)	90 (−1)	87.8 ± 0.2	88.6	0.9
7	55 (−1)	8 (0)	150 (1)	60.6 ± 2.3	59.7	0.9
8	75 (1)	8 (0)	150 (1)	87.8 ± 0.5	86.8	1.0
9	65 (0)	4 (−1)	90 (−1)	55.9 ± 0.6	55.4	0.5
10	65 (0)	12 (1)	90 (−1)	82.8 ± 0.1	81.4	1.4
11	65 (0)	4 (−1)	150 (1)	46.8 ± 0.4	48.2	1.4
12	65 (0)	12 (1)	150 (1)	84.8 ± 0.3	85.3	0.5
13	65 (0)	8 (0)	120 (0)	73.4 ± 0.1	72.6	0.1
14	65 (0)	8 (0)	120 (0)	72.5 ± 0.3	72.6	0.9
15	65 (0)	8 (0)	120 (0)	71.8 ± 0.5	72.6	0.8

^a^: Treatments were run in random order; ^b^: all analyses were conducted in triplicate and averaged; ^c^: (−1), (0), and (1) are coded levels.

**Table 2:** Analysis of variance for synthetic variables on response percent molar conversion.

Source	Sum of Squares	Degree of Freedom	*p > F ^a^*
*Linear*			
Temp. (°C) (*X*_1_)	1488.67	1	<0.0001
Time (h) (*X*_2_)	1996.49	1	<0.0001
Power (W) (*X*_3_)	5.43	1	0.1340
*Quadratic*			
Temp^2^	0.77	1	0.5312
Time^2^	135.2	1	0.0003
Power^2^	4.08	1	0.1821
*Interactions*			
Temp × Time	24.3	1	0.0129
Temp × Power	0.055	1	0.8641
Time × Power	30.91	1	0.0080
*Regression model*			
Model	3692.08	9	<0.0001
Residual	9.84	5	
Lack of fit	8.5	3	0.1972
Pure error	1.34	2	
Total	3701.92	14	
*R*^2^ = 0.997; Adj *R*^2^ = 0.992; C.V. % = 2			

^a^*p* > *F* = level of significance.

**Table 3:** Experimental and predicted values of the optimization conditions.

Independent Variable ^a^			Molar Conversion (%)		
*X*_1_	*X*_2_	*X*_3_	ActualValue ^b^	RSM Predict	RSM Deviation
74.6	11.1	150	93.8	94.6	0.8

^a^: Independent variable; *X*_1_: temperature (°C); *X*_2_: time (h) *X*_3_: power (W); and ^b^: mean of duplicate determinations.

## Data Availability

Not applicable.
